# Accessing mechanical thrombectomy treatment for stroke in England, Wales and Northern Ireland: the importance of the emergency pathway

**DOI:** 10.1136/emermed-2024-214134

**Published:** 2024-08-29

**Authors:** Christopher Price, Gary A Ford, Phil White, Martin James, Lisa Shaw

**Affiliations:** 1 Population Health Sciences Institute, Newcastle University Faculty of Medical Sciences, Newcastle upon Tyne, UK; 2 Medical Sciences Division, Oxford University Hospitals NHS Foundation Trust, Oxford, UK; 3 Translational and Clinical Research Institute, Newcastle University, Newcastle upon Tyne, UK; 4 Peninsula Applied Research Collaboration (PenARC), Royal Devon and Exeter NHS Foundation Trust, Exeter, UK

 Severe disability due to large vessel occlusion (LVO) stroke can be avoided by emergency mechanical thrombectomy (MT)^1^ but provision varies nationally and internationally according to the availability of specialist facilities. Across England, Wales and Northern Ireland, only 26 hospitals can offer treatment,^2^ and the majority of stroke patients are instead admitted to local non-MT hospitals. Here, eligible LVO cases must be quickly recognised through clinical assessment and brain imaging before transfer to the nearest MT-capable centre. Although this process offers a practical solution within the existing service infrastructure, cross-organisational pathways might impede access to time-critical interventions. As there has not yet been a population-level examination of whether transfer pathways influence access to MT, we used routinely collected data to retrospectively compare the treatment rates for patients first admitted to non-MT hospitals (where secondary transfers are necessary) relative to patients directly admitted to MT-capable centres in England, Wales and Northern Ireland.

For three consecutive years from 1 April 2020 to 31 March 2023, data were extracted from reports published by the Sentinel Stroke National Audit Programme describing the total number of admissions to non-MT hospitals and MT centres, and whether treatment was received.^2^ For each year, an OR was calculated to compare the proportion of patients receiving treatment via non-MT hospitals (ie, requiring secondary transfer) relative to MT centres (ie, direct admissions). It was assumed that LVO incidence was similar among patients presenting to all hospitals, formal bypass pathways were not in use and ambulance practitioners were not informally selecting patients with severe symptoms for direct conveyance to MT centres instead of nearer non-MT hospitals. As a retrospective examination of publicly available and anonymous aggregate data, ethical approval was not required.

During 2020–2021, 2021–2022 and 2022–2023, the proportion of total stroke admissions to non-MT hospitals remained stable at 72.1% (61 614/85 464), 71.6% (65 371/91 214) and 71.9% (65 574/91 162), respectively. Treatment activity data for all 3 years were available from 24 MT centres. Two small centres reporting 31 treatments were excluded because they did not accept transfers from non-MT hospitals and data from 2020 to 2021 were missing. The total population MT rate increased annually from 1.9% (1673/85 464) to 2.4% (2158/91 214) and 3.1% (2805/91 162), but the probability of receiving treatment was always significantly lower for patients first admitted to non-MT hospitals versus MT centres with ORs of 0.36, 0.44 and 0.47 (table 1).

**Table 1:** Treatment by mechanical thrombectomy (MT) per year according to route of admission

Year	Treatment of direct admissions to MT centres(MT/total admissions)	Treatment of secondary transfers from non-MT hospitals(MT/total admissions)	OR (95% CI) of receiving MT through secondary transfer versus direct admission
1 April 2020 to 31 March 2021	867/23 850 (3.6%)	806/61 614 (1.3%)	0.36 (0.33 to 0.39)
1 April 2021 to 31 March 2022	1024/25 843 (3.9%)	1134/65 371 (1.7%)	0.44 (0.40 to 0.48)
1 April 2022 to 31 March 2023	1277/25 588 (5.0%)	1528/65 574 (2.3%)	0.47 (0.43 to 0.50)

Despite yearly increases in the absolute volume of MT procedures being performed, a significant imbalance continues to exist between treatment of directly admitted patients and the much larger secondary transfer population. Providing equitable access to MT at the 10% population rate anticipated by national policy^3^ will require a specific focus on solutions to narrow this gap, and services should be aware that simply monitoring the overall MT rates or direct admission treatment numbers will not reflect the greatest unmet need.

One approach to improving MT access using the existing infrastructure would be expansion of direct admission footprints, but this is not feasible due to the large volume of patients involved, most of whom would not require MT.^4^ Previously, autonomous ambulance practitioner redirection of potentially eligible patients using a structured symptom assessment did not show a net improvement in health outcomes,^5^ and this approach has not been formally adopted by the NHS. However, ongoing research is evaluating a direct admission pathway for ambulance patients selected remotely by clinicians based at MT centres.^6^

It should be acknowledged that these aggregate results reflect three nations combined, and some regions will be more affected than others due to the size of direct admission footprints, hours of service operation, secondary transfer distances, maturity of local pathways and treatment eligibility of the underlying stroke population. The data collection interval started before many services had standardised their local MT referral processes, and the overall trend towards improving treatment rates is likely to reflect implementation of clearer emergency stroke pathways. However, clinical networks should continue to optimise MT access for all eligible patients by adopting a population perspective and evaluating current processes for LVO identification and transfer, whilst engaging in collaborative research to understand modifiable and non-modifiable reasons why provision varies between settings, such as differences in LVO incidence.
